# Complete Genome Sequence of Longicatena caecimuris Strain 3BBH23, Isolated from Healthy Japanese Feces

**DOI:** 10.1128/MRA.00282-21

**Published:** 2021-05-20

**Authors:** Yusuke Ogata, Mitsuo Sakamoto, Moriya Ohkuma, Masahira Hattori, Wataru Suda

**Affiliations:** a Laboratory for Microbiome Sciences, RIKEN Center for Integrative Medical Sciences, Yokohama, Japan; b Microbe Division/Japan Collection of Microorganisms, RIKEN BioResource Research Center, Tsukuba, Ibaraki, Japan; c PRIME, Japan Agency for Medical Research and Development (AMED), Tsukuba, Ibaraki, Japan; d Graduate School of Advanced Science and Engineering, Waseda University, Tokyo, Japan; University of Rochester School of Medicine and Dentistry

## Abstract

Here, we report the complete genome sequence of the Longicatena caecimuris strain 3BBH23, isolated from a healthy human Japanese feces sample. The genome is composed of a circular chromosome 3,103,757 bp long with a 38.3% GC content.

## Abstract (precis)

Here, we report the complete genome sequence of the Longicatena caecimuris strain 3BBH23, isolated from a healthy human Japanese feces sample. The genome is composed of a circular chromosome of 3,103,757 bp in length with a 38.3% GC content.

## ANNOUNCEMENT

Longicatena caecimuris was first isolated from the mouse gut, and the cells are rods, forming long filaments (1). Here, we report the complete genome sequence of Longicatena caecimuris strain 3BBH23, isolated from a healthy human Japanese feces sample. This is the first report of the complete genome sequence of this species.

The experiments were conducted in accordance with the RIKEN ethics committee (approval no. Tsukuba 27-1). A fecal sample (0.5 g) was suspended in 4.5 ml of prereduced phosphate-buffered saline (PBS). The diluted fecal sample was plated onto Brucella blood agar supplemented with 5 mg hemin and 0.5 mg menadione (per liter) for 2 to 4 days of incubation at 37°C under an H_2_/CO_2_/N_2_ (1:1:8, by volume) gas mixture. The grown colonies were screened by partial 16S rRNA gene sequencing as previously described (2) to identify and isolate Longicatena caecimuris strain 3BBH23. Strain 3BBH23 was then grown in 500 ml of Gifu anaerobic medium (GAM broth; Nissui) for 7 days at 37°C to prepare the genomic DNA. The DNA was extracted by enzymatic digestion as described previously (3). The DNA sequencing was performed with two platforms, Illumina MiSeq (2 × 300-bp paired ends) and PacBio Sequel, and the libraries were prepared following the manufacturer’s instructions using a TruSeq DNA PCR-free kit (target length, 550 bp) and SMRTbell template prep kit 2.0 (target length, 10 to 15 kbp), respectively, without DNA shearing. The MiSeq reads were trimmed and filtered with a >20 quality value (QV) using the FASTX-Toolkit v. 0.0.13 (hannonlab.cshl.edu/fastx_toolkit), and the Sequel reads were corrected using Canu v. 1.8 (4) with additional parameters as previously described (5). *De novo* hybrid assembly of both quality-checked reads was performed using Unicycler v. 0.4.8 (6), which contains a check of the generated contig for overlapping and circularization. Gene prediction and annotation of the generated contig was performed using the DDBJ Fast Annotation and Submission Tool (DFAST) v. 1.2.4 (https://dfast.ddbj.nig.ac.jp). Default parameters were used for all software unless otherwise specified.

We obtained a total of 1,133,680,423 bases from 1,895,705 filter-passed MiSeq paired-end reads with an average length of 299 bp and a total of 1,266,004,594 bases from 125,148 Sequel reads with an *N*_50_ value of 12,337 bp. The hybrid assembly of both reads generated a circular contig with a 773× coverage, corresponding to the *L. caecimuris* strain 3BBH23 chromosome.

The *L. caecimuris* strain 3BBH23 chromosome is 3,103,757 bp long with a 38.3% GC content and has 2,866 protein-coding genes, 44 tRNA genes, 5 5S rRNA genes, 5 16S rRNA genes, and 5 23S rRNA genes. The 16S rRNA sequence had the highest similarity to that of *L. caecimuris* strain PG-426-CC-2, with 99.79% identity in the NCBI rRNA/ITS database (24 February 2021).

### Data availability.

The complete genome sequence of *L. caecimuris* strain 3BBH23 was deposited in DDBJ/ENA/GenBank under accession no. AP024510, which is linked to the BioProject accession no. PRJDB11313, the BioSample accession no. SAMD00283801, and the DDBJ Sequence Read Archive (DRA) accession no. DRP007038.
